# A rare cause of deep vein thrombosis: inferior vena cava agenesis

**DOI:** 10.1590/1677-5449.202201262

**Published:** 2023-07-17

**Authors:** Pablo Del Canto Peruyera, Manuel Javier Vallina-Victorero Vázquez

**Affiliations:** 1 Hospital Universitario de Cabueñes - HUCAB, Servicio de Angiología y Cirugía Vascular, Gijón, España.

**Keywords:** inferior vena cava, agenesis, deep venous thrombosis, diagnosis, treatment, vascular congenital anomalies, veia cava inferior, agenesia, trombose venosa profunda, diagnóstico, tratamento, anomalias vasculares congênitas

## Abstract

Inferior vena cava agenesis is a rare condition and is often misdiagnosed. This anomaly is asymptomatic in the majority of cases and is usually diagnosed during imaging tests carried out for other purposes. The most frequent manifestation is deep vein thrombosis (DVT) in lower limbs and anticoagulation therapy is the most frequent treatment option. Other techniques such as thrombolysis and venous bypass are also described. We report two cases diagnosed at our institution during the last year, both of which presented with an episode of DVT. We opted for indefinite anticoagulation therapy and both patients remain asymptomatic, after 1 year of surveillance in the first case and 6 months in the second, with no new episodes of DVT. Although it is not a life-threatening anomaly, it is important to make an appropriate diagnosis and provide treatment to improve the symptoms and quality of life of these patients.

## Resumo

A agenesia da veia cava inferior é uma condição rara e, muitas vezes, mal diagnosticada. Essa anomalia é, na maioria dos casos, assintomática, sendo geralmente diagnosticada durante exames de imagem realizados com outras finalidades. A manifestação mais comum é trombose venosa profunda (TVP) em membros inferiores, e a terapia anticoagulante é a opção de tratamento mais frequente, embora outras técnicas, como trombólise e derivações venosas, também tenham sido descritas. Relatamos dois casos diagnosticados na nossa Instituição no último ano, ambos com episódio de TVP. O tratamento consistiu em anticoagulação por tempo indeterminado e, após 1 ano de acompanhamento no primeiro caso e 6 meses no segundo, ambos os pacientes permanecem assintomáticos, sem novos episódios de TVP. Embora não seja uma anomalia com risco de vida, é importante realizar diagnóstico e tratamento adequados para melhorar os sintomas e a qualidade de vida desses pacientes.

## INTRODUCTION

Agenesis of the inferior vena cava (IVC) is a rare vascular anomaly. The first case report by Wardrop Griffith dates back to 1891, in which drainage of the lower body was through the hepatic and azygos veins, entering directly into the atrium.^1^

Prevalence of IVC agenesis in the general population is estimated to be 0.3-0.6%.^2^ Among patients with other cardiovascular anomalies, IVC agenesis will be more frequent, reaching figures of 0.6-2%.^3^

Incidence is similar between Asian and European populations and is slightly higher in males (62%).^4^

Patients are usually asymptomatic and agenesis is diagnosed after imaging tests performed for other purposes.^2^ Deep vein thrombosis (DVT) of lower limbs is the most frequent manifestation.^5^

In lieu of the ethics committee, the statements of the Helsinki declaration were followed.

## CASE DESCRIPTION 1

The patient is a 24-year-old male with a 2 pack-year smoking history. He presents with lower limb pain and lumbar pain, edema and warmth of the right leg with muscle tenderness from the foot to the upper thigh. Distal pulses are palpable. Blood test shows Hb 10.9 g/dl and D-Dimer > 5000 ng/ml.

Suspecting DVT, lower limb Doppler ultrasound (DUS) is performed, showing images suggestive of DVT from popliteal to iliac veins.

**Figure 1:**
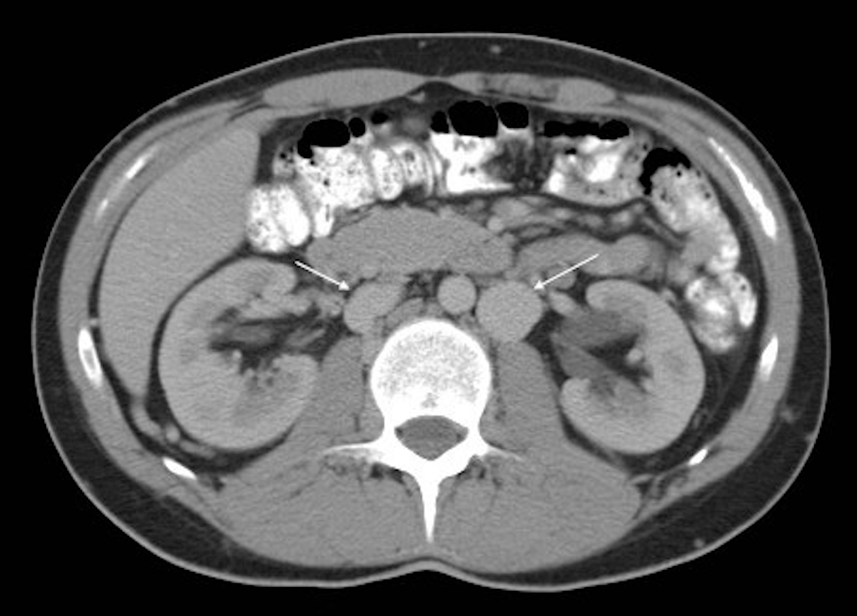
Azygos and hemiazygos system hypertrophy.

**Figure 2:**
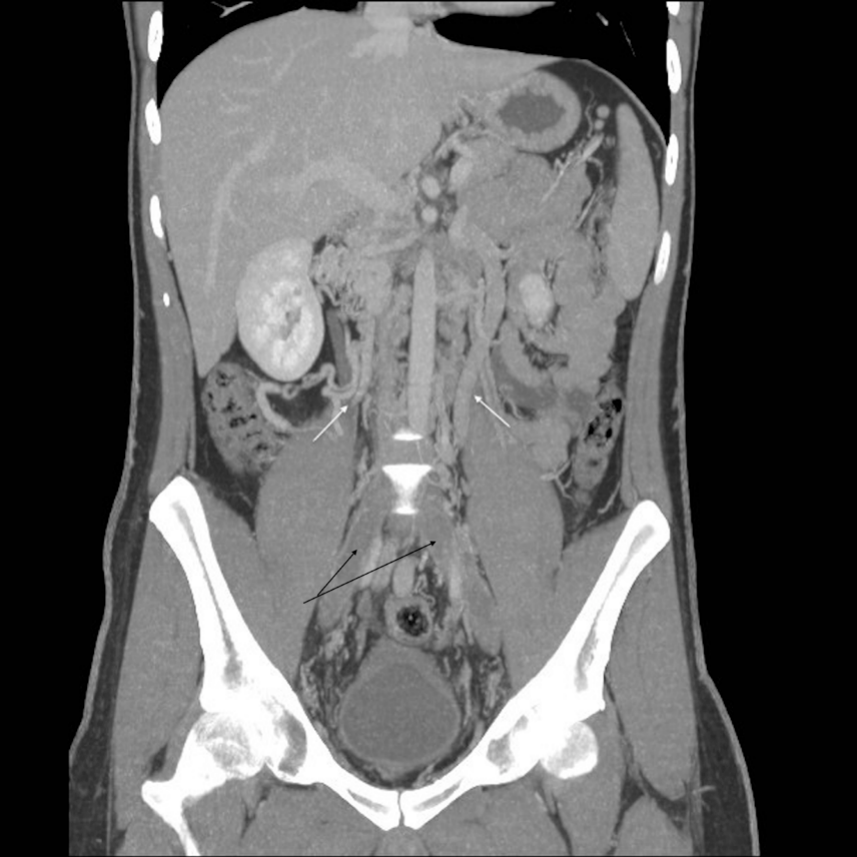
(White arrows) Azygos and hemiazygos system hypertrophy; (black arrows) thrombosed iliac veins.

After patient admission, we start anticoagulant treatment with low molecular weight heparin (LMWH) (enoxaparin 80mg/12 hours). In computed angiotomography (AngioCT) we observe IVC agenesis with large collateral circulation in lumbar and hemorrhoidal plexus, as well as azygos and hemiazygos system hypertrophy (Figure 1 and Figure 2).

Thrombophilia screening shows positive lupus anticoagulant and IgM anticardiolipin antibody levels of 26 MPL U/ml. This first measurement was taken within 2 days of starting anticoagulation with LWMH. A second measurement to confirm or rule out a possible coagulation disorder was not performed at 12 weeks due to patient refusal, so a definitive diagnosis was not possible.

We decided on indefinite oral anticoagulation with acenocoumarol and after 1 year of surveillance the patient remains asymptomatic with no new episodes of DVT.

## CASE DESCRIPTION 2

A 21-year-old male presents with pain and swelling of the right leg. He presents hypertension secondary to a nonfunctional left kidney due to left renal vein thrombosis during childhood and he also had an episode of DVT in the right leg 5 years ago. On physical examination we observe large collateral circulation at the right groin. Blood test shows D-Dimer > 5000 ng/ml. DUS confirms a new episode of DVT with thrombosis from the common femoral to the popliteal vein.

**Figure 3:**
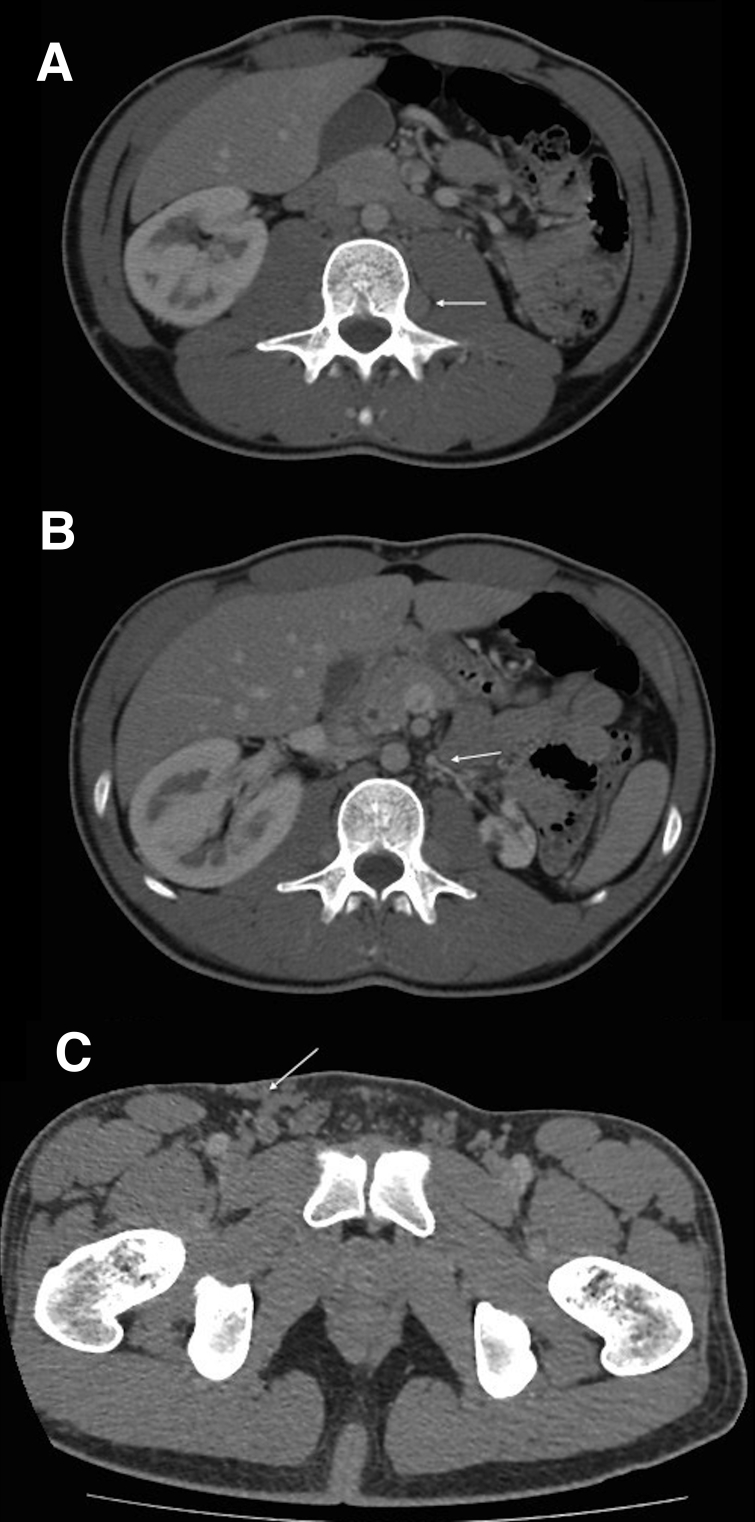
(A) Left iliac vein ascending along the back of the psoas; (B) hypoplastic left renal vein; (C) varicose veins connecting with the superficial femoral vein at the right groin.

As this is the second episode of DVT, we decide to perform an Angio-CT. This Angio-CT shows infrarenal IVC agenesis with common iliac veins running through different routes: on the right side adjacent to the aorta and on the left side ascending along the back of the psoas with a paravertebral course and final drainage into the azygos and hemiazygos system; varicose veins are also observed connecting with the right femoral vein (Figure 3).

Thrombophilia screening shows no alterations, with a minimal increase in homocysteine levels (17.2 mcmol/L).

We start anticoagulant treatment with LMWH (enoxaparin 80 mg/12 hours) before moving on to oral anticoagulation with acenocoumarol.

After 6 months of anticoagulant therapy and elastic compression stockings the patient remains asymptomatic.

## DISCUSSION

The IVC is formed between weeks 4 and 8 of embryonic development from 3 pairs of veins: the cardinal, subcardinal, and supracardinal veins.^6^ The cardinal veins are the first to develop and are predominant until the 6th week of gestation. They will not become part of the IVC in adults, but abnormalities in them may give rise to anomalies in the IVC.^7^ Subcardinal veins will be the predominant ones by the 7th week and the right subcardinal vein will form the suprarenal segment of the IVC.^7^ Supracardinal veins start developing during the 6th week, but it will not be until the 8th week that they take on leading roles, extending above the diaphragm to form the azygos and hemiazygos veins, and the right supracardinal vein will eventually lead to the infrarenal segment of the IVC.^7^ Any failure during these 4 weeks of embryonic development will lead to different anomalies of the IVC, the most prevalent of which are the retroaortic or circumaortic left renal vein, double IVC, left IVC, azygos and hemiazygos continuation, and IVC agenesis.^6^

Suprarenal segment agenesis is the most common form of IVC agenesis, while infrarenal segment agenesis or agenesis of the entire IVC are rare (only 6% of all cases of agenesis).^5^

Two theories about the IVC agenesis are described. One is a failure during embryonic development and in this case it can be associated with malformations in other locations such as the heart, spleen (asplenia and polysplenia), and bowel.^6^ The other theory would explain the absence of IVC during adulthood as a result of a thrombosis during the perinatal period, which would cause abnormal development.^8^

One case that would support the theory of perinatal thrombosis was published by Wax et al.,^9^ describing a patient who underwent two cardiac catheterizations at 4 months of age, with no evidence of anomalies in the venous anatomy. However, IVC agenesis was observed in Angio-CT performed at 26 years of age due to pelvic varicosities.^9^

Many cases of IVC agenesis occur in patients with alterations of thrombophilia studies, as in the first case we describe where titers of IgM anticardiolipin antibodies (26 MPL U/ml) and lupus anticoagulant were identified, although a final diagnosis was not made; the association of cava agenesis, DVT, and thrombophilia, although described, has not been fully tested.^10^ In the event that this association exists, we should ask ourselves whether this is a true association or if it is actually a cause-effect relationship, supporting the theory of IVC thrombosis during perinatal period as cause of future IVC agenesis, by which infants with thrombophilic disorders could suffer IVC thrombosis during the perinatal period causing atrophy that would then be identified as IVC agenesis in adulthood.

IVC agenesis is usually asymptomatic and often diagnosed after imaging tests performed for other purposes.^2^ In case of symptoms, DVT will be the main manifestation. Patients with IVC agenesis present a lifelong risk of DVT of around 10%.^5^

In the absence of the IVC, compensatory mechanisms are formed, especially in 4 systems:^11^ the gonadal venous system, paravertebral venous plexus, hemorrhoidal plexus, and superficial veins.

DVT often occurs after intense exercise because the collateral veins developed are unable to deal with all the venous drainage produced during exercise, causing venous stasis and thereby facilitating thrombus formation.^2^ DVT are bilateral in up to 50% of cases.^3^ Lumbar vein thrombosis can be present causing lumbar pain, as in the first case we describe.^2^ Although the risk of DVT is increased in these patients, the rate of pulmonary embolism (PE) is low (9%).^11^ To reach the pulmonary circulation, thrombus would have to cross a tortuous route through collateral veins, being retained therein in most cases.^11^

Other symptoms that may result are: venous claudication,^12^ hematuria caused by bladder varicosities,^13^ lumbar pain due to venous collaterals in the vertebral and paravertebral plexus, causing compression of the thecal sac and at the output of the nerve roots,^14^ cauda equina symptoms such as bladder or bowel dysfunction and lower limb weakness,^15^ visceral compressions by the gonadal venous system,^16^ venous ulcers due to deep venous insufficiency,^17^ and venous aneurysm.^16^

Diagnosis is made after Angio-CT, magnetic angioresonance, or phlebography, of which Angio-CT offers the best cost-effectiveness ratio and easier access.^18^ DUS would not be an appropriate test to assess IVC malformations.^18^

Different therapeutic options have been described, although most authors agree that anticoagulation is the one that offers the best results.^19^ Regarding the duration of anticoagulation, there are no conclusive studies to opt for a specific duration, however long-term anticoagulation has the greatest acceptance, especially given the high rate of DVT recurrence when anticoagulation is stopped.^20^

Thrombolysis has also been described, but this technique is currently obsolete because of high recurrence rates.^17^

There are reported cases in which patients were treated by open surgery with a venous bypass with successful results, but it is not a widely used treatment option because of the limited experience with the technique and the lack of long-term results.^2^ Angioplasty and stenting are also described, with varied results.^2^

Although IVC agenesis is a rare anomaly, we should consider it when faced with a young patient (without classical cardiovascular risk factors) who presents with DVT. Among patients younger than 30 years with an episode of DVT, IVC agenesis has been observed in up to 5% of cases.^10^

Angio-CT is the recommended imaging test for diagnosis, offering a better cost-effectiveness ratio and easier access.

Regarding treatment, our recommendation is indefinite anticoagulation. The risk factor for DVT will not disappear over time and will always be a threat for a new episode.

Patients should avoid overloading the system of venous collaterals that have developed, avoiding venous stasis and reducing the likelihood of thrombosis; in concrete terms, we refer to intense physical exercise.

In the same way, they should avoid any other cardiovascular risk factor (tobacco and oral contraception). Compression stockings will be useful for decreasing symptoms of venous hypertension on lower limbs.

Although IVC agenesis is not a life-threatening disease, it may cause great discomfort and loss of quality of life due to repeated episodes of DVT and lower limb venous insufficiency; diagnosis will be very relevant in cases in which abdominal surgery is planned. For all these reasons, a proper study and treatment will be essential for preventing future episodes and improving patients’ quality of life.
